# Validating virtual reality for time perception research: Virtual reality changes expectations about the duration of physical processes, but not the sense of time

**DOI:** 10.3758/s13428-023-02201-6

**Published:** 2023-09-26

**Authors:** Johanna Bogon, Julian Högerl, Martin Kocur, Christian Wolff, Niels Henze, Martin Riemer

**Affiliations:** 1 Media Informatics Group, University of Regensburg, Regensburg, Germany; 2 Digital Media, University of Applied Sciences Upper Austria, Hagenberg, Austria; 3 Biological Psychology and Neuroergonomics, Technical University Berlin, Berlin, Germany

**Keywords:** Time perception, Time production, Virtual reality

## Abstract

Immersive virtual reality (VR) provides a versatile method for investigating human time perception, because it allows the manipulation and control of relevant variables (e.g., the speed of environmental changes) that cannot be modified in the real world. However, an important premise for interpreting the results of VR studies, namely that the method itself does not affect time perception, has received little attention. Here we tested this assumption by comparing timing performance in a real environment and a VR scenario. Participants performed two timing tasks, requiring the production of intervals defined either by numerical values ("eight seconds") or by a physical process (“the time it takes for a bottle to run out when turned over"). We found that the experience of immersive VR exclusively altered judgments about the duration of physical processes, whereas judgments about the duration of abstract time units were unaffected. These results demonstrate that effects of VR on timing performance are not driven by changes in time perception itself, but rather by altered expectations regarding the duration of physical processes. The present study validates the use of VR in time perception research and strengthens the interpretation of changed timing behaviour induced by manipulations within VR.

In recent years, technical developments in virtual reality (VR) applications and human-machine interactions have been immensely fruitful for the investigation of human perception and behaviour (Dechant et al., 2017; Diersch and Wolbers, 2019; Kocur et al., 2022, 2020; Matamala-Gomez et al., 2019; Turbyne et al., 2021). In the field of human time perception, recent studies highlight the importance of using realistic stimuli, embedded within naturalistic environments (Boltz, 2005; Brunec et al., 2017; Maaß et al., 2022; Riemer et al., 2021; Schlichting et al., 2018; Tobin et al., 2010; van Rijn, 2018). Following this approach, manifold influences of environmental aspects like room size and general appearance of the surroundings have been confirmed (Aeschbach et al., 2022; Riemer et al., 2018). In order to systematically manipulate these factors in controlled experiments, VR represents a pivotal and promising methodology (Brookes et al., 2020; Knierim et al., 2020; Landeck et al., 2020, 2023; Read et al., 2021). However, an essential premise for the general comparability between timing behaviour in the real world and in VR settings has received little attention. In experiments employing VR techniques it often is implicitly assumed that VR itself does not induce changes in temporal processing. Although there is some evidence that certain changes in VR can alter the perception of time (e.g., by manipulating the speed of natural events like sunrise or sunset; Landeck et al., 2020: Schatzschneider et al., 2016), it is unclear to date whether the feeling of immersion that is induced by increasingly sophisticated VR techniques itself has an effect on time perception (Read et al., 2021). This information is absolutely necessary to interpret the results, and it is an essential basis for implications drawn from studies using VR techniques. Closing this gap is the aim of the present study.

There are two main reasons why immersive VR itself could alter the perception of time. First, it is known that motivational states as increased attention and emotional arousal affect time perception (Block et al., 2010; Droit-Volet and Gil, 2009; Droit-Volet and Meck, 2007). Interesting or arousing stimuli are perceived as temporally longer than uninteresting or trivial ones (Angrilli et al., 1997; Mella et al., 2011; Palumbo et al., 2014; Schiffman and Bobko, 1974). Given that for most people VR is less familiar and therefore potentially more interesting than the real world (at least for a certain period), it is reasonable to assume that the associated arousal results in a lengthening of experienced durations (Mullen and Davidenko, 2021). Similarly, van der Ham et al. (2019) suggest that a time compression effect in VR is mediated by the presented emotional content. Second, the immersiveness of increasingly sophisticated VR techniques can induce a feeling of dissociation from the real world and/or the own body (Aardema et al., 2010; Lenggenhager et al., 2007; Schubert et al., 2001; Witmer and Singer, 1998), and it is known that such dissociative experiences can alter the sense of time (Craig, 2009; Di Lernia et al., 2018; Vohs and Schmeichel, 2003; Wackermann et al., 2008; Wittmann, 2009).

The question as to whether VR itself alters the perception of time is related to a further issue about the interpretation of VR effects on time judgments: In many studies, VR techniques have been used to manipulate temporal information via external *zeitgeber* (e.g., delayed visual feedback for bodily movements, or increased speed of diurnal phases), and in most of them, an effect on timing performance could be confirmed (Bansal et al., 2019; Schatzschneider et al., 2016). However, these effects can be explained in two fundamentally different ways. First, by changes in basic timing mechanisms, meaning that time itself is indeed perceived differently. A second possibility is that basic timing mechanisms are unaltered and that the effects instead are based on altered temporal expectations regarding the duration of physical events. According to this assumption, people do not perceive time as running faster, they just learn that in this specific VR setting a “day” is shorter than usual, or objects move slower than in reality (it is one of the powerful advantages of VR that programmers can manipulate these aspects). Obviously, these two interpretations lead to very different conclusions regarding timing processes.

To disentangle the effects of VR on basic timing mechanisms from those based on recalibrated expectations, we compared the performance in two different time perception tasks in the real world and in two VR conditions (one with normal and one with decelerated feedback regarding own body movements). The first task requires the production of time intervals indicated by abstract numerical units (e.g., “8 s”), and the other task requires the production of time intervals corresponding to specific physical processes (e.g., “the time it takes for a filled bottle to run out when turned over”). If the experience of VR changes timing mechanisms on a basic level, both types of tasks should be affected. Eight seconds should be over- or underestimated to the same degree and in the same direction as the duration of a bottle running out. On the other hand, if VR only induces altered expectations with respect to the duration of concrete physical processes (“time doesn’t slow down, it’s just that things take more time”), then only the duration of the physical process should be biased (especially in the VR condition with decelerated movement feedback), while the production of "eight seconds" should not differ between VR and the same set-up in the real world.

## Method

### Participants

We recruited 37 students from the University of Regensburg and the local community who participated voluntarily or for course credits to achieve an effective sample size of at least 34 participants after participant exclusion. With an alpha level set at .05, this sample size ensured a power of 1 - $\beta$ > .85 for detecting the relevant task type x environment interaction effect with an effect size of $\eta _{p}^{2}$ = .15, and a power of 1 - $\beta$ > .80 for detecting ratio differences in pairwise comparisons of environment conditions in each task with an effect size of Cohen’s *d* > 0.5 (calculated with MorePower Version 6.0.4; Campbell and Thompson, 2012). One participant had to abort the experimental procedure due to cybersickness. Data of one further participant were excluded because their produced intervals were more than three interquartile ranges above the third quartile of the sample distribution. The final sample consisted of 35 participants (age *M* = 24.2 years, *SD* = 5.5; 12 self-identified as female, 23 as male; 30 right-handed, 5 left-handed). Before starting the experiment, all participants provided informed, written consent.

### Apparatus

**Fig. 1:**
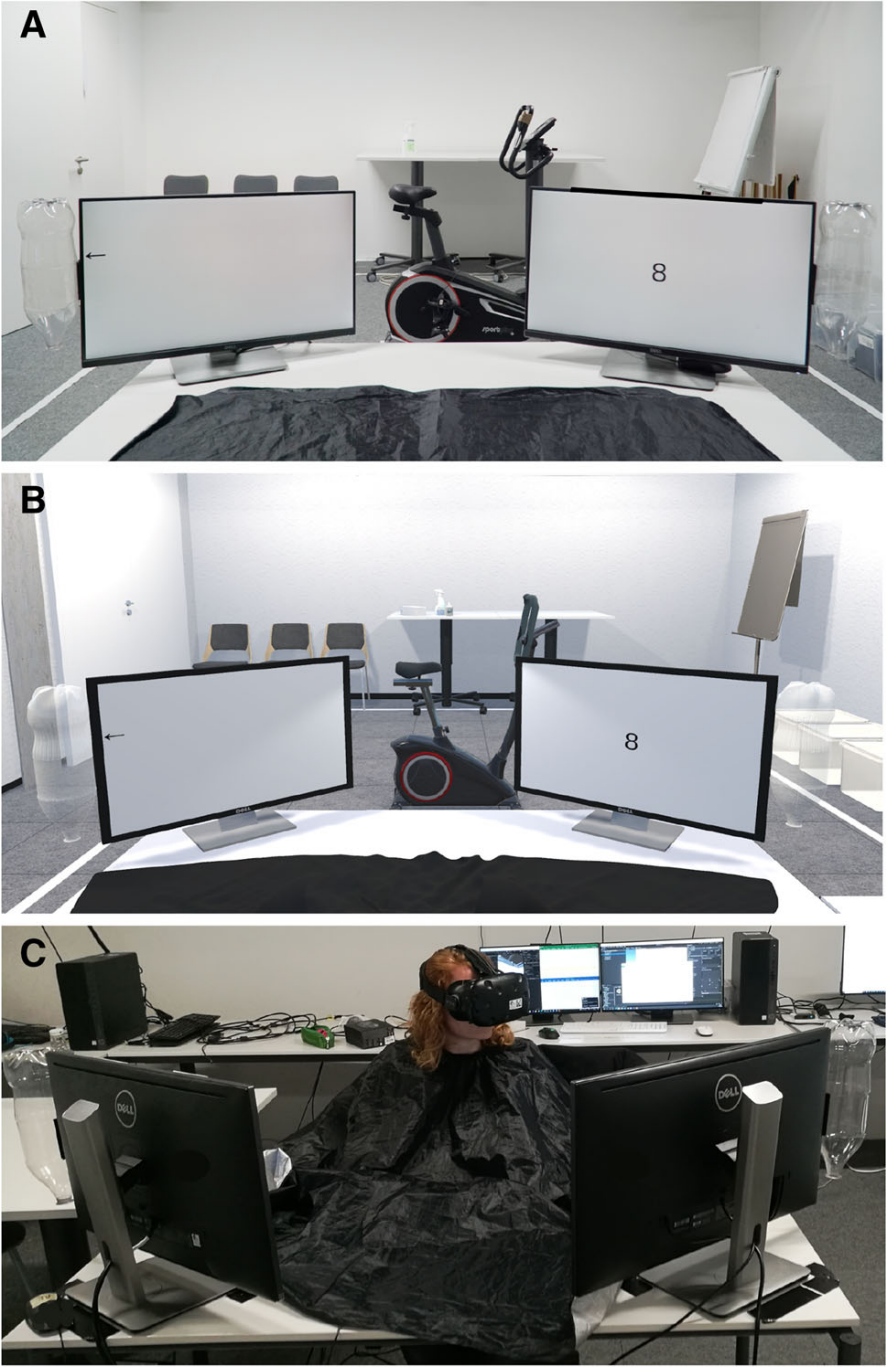
Conditions and experimental setup. Panel A: Real environment condition (RE). Panel B: Virtual reality conditions (VR_bio_ and VR_slow_). Panel C: Experimental setup

The experimental setup is illustrated in Fig. 1. The participant sat at a table with two 27-inch Dell U2715H monitors with a resolution of 2560 × 1440 and a refresh rate of 60 Hz. Both displays were placed at an angle of 45 degrees to the left and right of the participant with a viewing distance of approximately 90 cm (see Fig. 1C). A standard bottle (well-known brand, capacity: 2 ls) without a lid and without a cover was attached to one side border of each monitor (left border of left monitor and right border of right monitor) with the bottle opening facing down. To increase comparability of virtual and real environments, participants wore a black barber cape that covered hands and arms. Responses in the time perception tasks were given by pressing the button of a standard mouse.

The virtual replica of the real environment and the task procedures were designed and implemented using the game engine Unity 3D (version 2020.3.25f1, https://unity.com/). To present the virtual environment and track head movements, an HTC vive head-mounted display (HMD) was used with a horizontal field-of-view of 100° and a resolution of 1080 x 1200 pixels per eye displayed with a refresh rate of 90 Hz.

### Tasks and stimuli

In the numerical-unit task, participants had to produce one of four standard intervals (1, 2, 4 and 8 s). Each trial began with a fixation cross that was displayed for 4000 ms. Then, the standard interval was displayed in the middle of a gray screen as an abstract numerical value (number 1, 2, 4 or 8).

In the physical-process task, participants were asked to produce the time it takes for a filled bottle to run out when turned over. In each trial, after a fixation cross that was displayed for 4000 ms, a black arrow pointing to the bottle fixed at the monitor’s edge was displayed at one of four vertical positions at the side of the gray screen (cf. Fig. 1A). The positions indicated filling heights that corresponded to 1, 2, 4 and 8 s pouring duration.

In both tasks, target intervals were produced by pressing the mouse button twice, the first press indicating the start and the second press indicating the end of the produced interval. After the first button press, the color of the screen changed to green until the second button press.

**Fig. 2:**
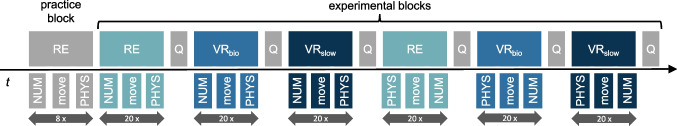
Experimental procedure. After a practice block with four trials per task, each participant underwent all three environment conditions (RE, VR_bio_, VR_slow_) twice, resulting in six experimental blocks. Each block comprised 20 numerical-unit trials (NUM) and 20 physical-process trials (PHYS). Numerical-unit trials were presented on the left monitor and physical-process trials on the right monitor (or vice versa). Numerical-unit trials and physical-process trials alternated, so that participants were continuously forced to turn their head from one side to the other before each trial (move). This ensured that the participants experienced the difference between the VR_bio_ and VR_slow_ conditions. The side at which the tasks were presented (e.g., numerical-unit trials at the left and physical-process trials at the right monitor) was held constant within the first three and the last three experimental blocks, but changed between block three and four. Each block was followed by questionnaires (Q). The order of the three environment conditions was held constant within participants but was balanced between participants using a Latin square

### Environment conditions

In the real environment (RE) condition, participants performed the time perception tasks in the real laboratory environment (see Fig. 1A) without wearing the HMD. In the virtual reality conditions (VR_bio_ and VR_slow_), participants performed the tasks in a virtual version of the laboratory that was presented via the HMD (see Fig. 1B). The two VR conditions differed with regard to the speed of the translation of the tracked head movements to the visual display of the HMD. In the VR_bio_ condition, head movements were translated in real time. In the VR_slow_ condition, we manipulated this translation to provide participants with the sensation that they were moving slower than intended. We aimed to create a slow-motion effect without delaying the users’ head motion as this could be interpreted as an input lag or technical issue. In line with Rietzler et al. (2017), we therefore implemented the VR_slow_ condition using an algorithm that always reacted to the users’ head motion without using a delay but still creating the impression of slower motion. We achieved this by a linear interpolation between the HMD’s actual position and rotation and a virtual camera’s position and rotation that was reduced by a constant factor *t* = 0.07 for every frame. As participants perceived the virtual environment from the perspective of the virtual camera, their virtual head movement was slower compared to the actual movement of the HMD:

1 $$\begin{aligned} Pos_{new} = Pos_{act} + t(Pos_{HMD} - Pos_{act}) \end{aligned}$$

2 $$\begin{aligned} Rot_{new} = Rot_{act} + t(Rot_{HMD} - Rot_{act}) \end{aligned}$$

To test which value for factor *t* produces a feeling of slowing down but does not have unpleasant consequences (e.g., dizziness, headache, or nausea), we piloted the VR_slow_ condition with varying values for *t*, ranging from *t* = 0.01 to *t* = 0.1 with seven participants that did not participate in the main study. Based on these participants’ verbal feedback, we set the factor *t* to 0.07, i.e., visual feedback for head movements in the VR_slow_ condition was approximately half as fast as the normal speed in the VR_bio_ condition.

### Questionnaires

We assessed the feeling of presence in the VR conditions, using the Igroup Presence Questionnaire (IPQ; Schubert et al., 2001). Furthermore, to control for any negative effects of the VR conditions, we asked five questions regarding symptoms of simulator sickness (Kennedy et al., 1993) after each condition. These questions accounted for fatigue, general discomfort, headache, and dizziness.

### Procedure

The experimental procedure is illustrated in Fig. 2. Each participant underwent all three environment conditions (RE, VR_bio_, VR_slow_) twice, resulting in six experimental blocks. Each block comprised 20 numerical-unit trials and 20 physical-process trials with each of the four standard intervals occurring five times per task. Numerical-unit trials were presented on the left monitor and physical-process trials on the right monitor (or vice versa). Numerical-unit trials and physical-process trials alternated, so that participants were continuously forced to turn their head from one side to the other before each trial. This ensured that the participants experienced the difference between the VR_bio_ and VR_slow_ conditions. At the end of each trial, a short acoustic cue was presented to remind the participant to move the head to the other monitor. The side at which the tasks were presented (e.g., numerical-unit trials at the left and physical-process trials at the right monitor) was held constant within the first three and the last three experimental blocks, but changed between block three and four. The initial assignment of tasks and side (during the first three blocks) was counterbalanced across participants. Each block was followed by five questions concerning simulator sickness. After the second VR_bio_ and the second VR_slow_ block, participants additionally completed the IPQ. After the last block, participants were asked whether they noticed any movement transition differences in the VR conditions (*"In some of the virtual reality conditions, head movements were slower than usual. Did you notice this?"*) and whether the transmission in the last condition was slow or normal (*"Were the movements in the last virtual reality condition slow or normal? You may also guess."*). The order of the three environment conditions was held constant within participants, but was balanced between participants according to a Latin square.

Before the actual experiment, each participant emptied a bottle of water three times (same model as in the experimental setup) to get a feeling for the duration of this process. For both tasks, four practice trials were performed in the real environment.

**Fig. 3:**
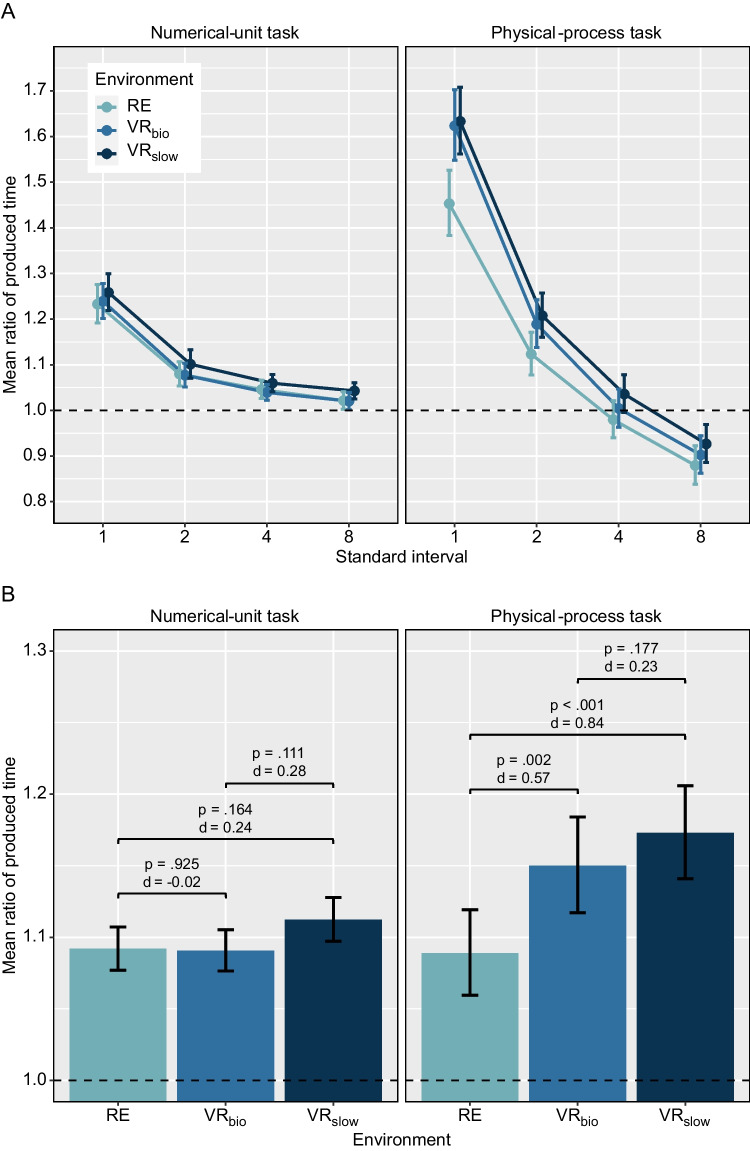
Mean ratios between produced time and standard intervals. Ratios greater than 1 indicate a contraction of perceived time and ratios below 1 indicate a dilation of perceived time. Mean ratio values are derived by back-transforming the mean ratio_log_ values ($ratio = e^{ratio_{log}}$) that were entered into the analyses. Error bars represent standard errors. Panel A: Mean ratios are shown as a function of task type, environment, and standard interval. Panel B: Mean ratios are shown as a function of task type and environment. For each pairwise comparison, corresponding *p*-values and effect sizes (*Cohens’s d*) are reported

### Statistical analyses and design

A complete analysis script and the raw data can be found at OSF (https://osf.io/gmrfq). Produced intervals of both tasks were standardized by calculating ratio scores between produced intervals and standard intervals. Ratios greater than 1 indicate a contraction of perceived time and ratios below 1 indicate a dilation of perceived time. Since the distribution of the ratio scores was left skewed, we log-transformed the ratio scores before statistical testing. We refer to the transformed variable as ratio_log_. To obtain corresponding interpretable non-standardized effect sizes, we additionally report mean ratio values that are derived by back-transforming the mean ratio_log_ values ($ratio = e^{ratio_{log}}$). Only extreme outliers, defined as ratio_log_ scores below -2 (0.02% of all trials) or exceeding three times the standard deviation of the respective individual cell means (none of all trials exceeded this criteria), were excluded from further analysis.

Data were analysed in R (version 4.2.3, R Core Team, 2023) using repeated measures ANOVAs and *t*-tests (R package *rstatix* version 0.7.2; Kassambara, 2023). Effects with violations of sphericity were Greenhouse-Geisser corrected and are reported with corresponding $\varepsilon$ estimates. To allow evaluation of potential null effects, we additionally conducted Bayesian *t*-tests for each pairwise comparison using default prior scales provided by the *BayesFactor* R package (version 0.9.12$-$4.4, Morey and Rouder, 2022; see also Rouder et al., 2009).

For analyzing the effect of the environment conditions on time perception, we used a 2 × 3 × 4 design, including the within-subjects factors task type (numerical-unit, physical-process), environment (RE, VR_bio_, VR_slow_) and standard interval (1, 2, 4, and 8 s) with ratio_log_ as dependent variable.

## Results

### Timing performance

Back-transformed mean ratio_log_ scores as a function of task type, environment, and standard interval are illustrated in Fig. 3A. The results of the 2 × 3 × 4 within-subjects ANOVA on the ratio_log_ scores are listed in Table 1. Our analysis revealed significant main effects for environment, *F*(2, 68) = 8.02, *p* = .001, $\eta _{p}^{2}$ = .19, and standard interval, *F*(1.17, 39.73) = 92.88, *p* < .001, $\eta _{p}^{2}$ = .73, $\varepsilon$ = .389. In addition, we found interaction effects between the factors task type and environment, *F*(2, 68) = 9.17, *p* < .001, $\eta _{p}^{2}$ = .21, and between task type and standard interval, *F*(1.25, 42.51) = 42.51, *p* < .001, $\eta _{p}^{2}$ = .55, $\varepsilon$ = .417.

**Table 1:** Statistics of the 2 x 2 x 4 ANOVA

Effect	*DF*_*n*_	*DF*_*d*_	*F*	*p*	$\eta _{p}^{2}$	$\varepsilon$
Task type (T)	1	34	1.14	.293	.03	
Environment (E)	2	68	8.02	.001	.19	
Standard interval (S)	1.17	39.73	92.88	<.001	.73	.389
T x E	2	68	9.17	<.001	.21	
T x S	1.25	42.51	42.35	<.001	.55	.417
E x S	3.51	119.23	2.45	.058	.13	.584
T x E x S	4.25	144.64	2.26	.062	.14	.709

*Note*. The ANOVA examined the logarithmised ratios between produced time and standard intervals (ratio_log_) as a function of the three within-subject factors task type (numerical-unit, physical-process), environment (RE, VR_bio_, VR_slow_) and standard interval (1, 2, 4, and 8 s). Effects with violated sphericity assumption have been corrected by the Greenhouse-Geisser method. For these effects the corresponding $\varepsilon$ estimate for the sphericity test is additionally reported

To further analyse the relevant interaction between task type and environment, we conducted separate one-way ANOVAs with the factor environment and pairwise *t*-tests on the ratio_log_ scores (averaged over standard intervals) for both tasks (Fig. 3B). In the numerical-unit task, the environment conditions did not significantly affect time judgments, *F*(2, 68) = 1.56, *p* = .218, $\eta _{p}^{2}$ = .04. Neither the VR_bio_ condition, *t*(34) = 0.09, *p* = .925, *d* = $-$0.02, 95% CI[$-$0.35, 0.32], nor the VR_slow_ condition, *t*(34) = 1.42, *p* = .164, *d* = 0.24, 95% CI[0.10, 0.57], differed significantly from the RE condition with regard to the ratio_log_ scores. For the RE vs. VR_bio_ comparison, a Bayes factor of BF_01_ = 5.49 indicated that the data are 5.49 times more likely under the null hypothesis that postulates identical ratio_log_ scores between conditions than under the alternative hypothesis that postulates a difference between ratio_log_ scores between conditions. For the RE vs. VR_slow_ comparison, the data are 2.19 times more likely under the null hypothesis than under the alternative hypothesis (BF_01_ = 2.19). However, in the physical-process task, there was a significant main effect of environment, *F*(2, 68) = 12.99, *p* < .001, $\eta _{p}^{2}$ = .28. In both VR conditions, ratio_log_ scores differed significantly from the RE condition. In the VR_bio_ condition, ratio_log_ was on average 0.05 units higher than in the RE condition, *t*(34) = 3.40, *p* = .002, *d* = 0.57, 95% CI[0.21, 0.93], BF_01_ = 0.05. The back-transformed ratio means differed by 0.06 units which would correspond to a relative contraction of perceived time by 6% (Fig.  3B). In the VR_slow_ condition, ratio_log_ was on average 0.07 units higher than in the RE condition, *t*(34) = 4.98, *p* < .001, *d* = 0.84, 95% CI[0.45, 1.22], BF_01_ < 0.01. This would correspond to a relative contraction of perceived time by 8% (Fig. 3B). Ratio_log_ scores did not differ significantly between the VR_bio_ and the VR_slow_ conditions, *t*(34) = 1.38, *p* = .177, *d* = 0.23, 95% CI[$-$0.10, 0.57], BF_01_ = 2.32.

### Questionnaire data

Thirty-one of 35 participants (89%) noticed transmission differences between the VR conditions, and 28 of them (90%) correctly identified the relative movement speed during the last VR condition.

The IPQ was analyzed to control for differences between the VR conditions. *t*-tests for each subscale of the IPQ (spatial presence, involvement, experienced realism) revealed a significant difference only for spatial presence, *t*(34) = 2.67, *p* = .012, *d* = 0.45, 95% CI[0.10, 0.80], with higher scores in the VR_bio_ condition compared to the VR_slow_ condition (4.2 vs. 3.9).

## Discussion

In the current study we aimed at answering a fundamental question regarding the perception of time in virtual reality (VR) setups. VR-induced changes in timing behaviour have been frequently reported (Bansal et al., 2019; Lugrin et al., 2019; Mullen and Davidenko, 2021; Schatzschneider et al., 2016), but it is unknown to date whether these changes are based on an inherently altered sense of time induced by VR, or alternatively on altered temporal expectations regarding the duration of physical processes occurring in virtual versus real environments. We tackled this question by comparing the performance in two time production tasks, one of which required the production of time intervals presented in abstract numerical units, and the other one requiring the production of time intervals related to a physical process that should be imagined in the real and virtual environments. Our reasoning was that, if VR induces a genuine shift in the perception of time itself, then the performance in both of these tasks should differ between real and virtual environments. In contrast, if VR only alters expectations regarding the duration of specific physical processes (e.g., water flows more slowly in VR, but time flow is unchanged), then behavioural changes should be observable exclusively in the physical-process task and not in the numerical-unit task.

Our results are in favor of the latter hypothesis. Both VR conditions led to an increase in the produced intervals in the physical-process task, while the produced intervals in the numerical-unit task were not statistically different from the RE condition.

This result provides a new perspective on alterations in timing behaviour induced by immersive VR (Bansal et al., 2019; Lugrin et al., 2019; Mullen and Davidenko, 2021; Schatzschneider et al., 2016) and offers an alternative interpretation: The VR-induced changes in timing behaviour might not be caused by a change in the perception of time per se (in the sense that time is perceived as running faster or slower), but instead by expectations about how the VR works. If we are asked to estimate the time it takes for a stone to fall from our hand to the ground, we would probably give different answers depending on whether we imagine this stone-falling scenario happening on the earth or on the moon (i.e., under conditions of less gravity). It would be wrong to conclude from this observation that we believe time is running slower on the moon, or that we perceive time running slower during our imagination of being on the moon. The different estimations regarding the temporal interval are merely caused by our knowledge about gravity differences and an assumption of how this affects physical processes.

The results of the present study question the interpretation that VR influences the genuine perception of time, thereby validating the utility of VR techniques as a tool to investigate the impact of environmental factors on time perception. In recent years, VR has been advocated as a powerful method for investigating changes in time perception (Brookes et al., 2020; Knierim et al., 2020; Landeck et al., 2020, 2023; Read et al., 2021). According to the logic that an assessment tool is only valid as long as it does not itself affect the object under investigation, the present study demonstrates that this is the case for the use of VR and time perception.

In line with this view, many of the studies comparing the performance in a specific task during an immersive VR versus a non-immersive Desktop condition did not find differences in temporal judgments as long as the judgments referred to numerical time units (Lugrin et al., 2019; Mallam et al., 2020; Schatzschneider et al., 2016; van der Ham et al., 2019). Mullen and Davidenko (2021) report an initial underestimation of time in VR (relative to a Desktop condition), but they also found evidence for that this effect is driven by the novelty of the VR experience. Similarly, van der Ham et al. (2019) compared time estimation performance in a real-life cinema situation with an analogue VR version of the same scenario. They found no difference between real-life and VR, but reported that time estimates were influenced by the emotional content of video clips presented in the real versus the virtual cinema. Accordingly, the authors argue that an effect of VR on time perception is driven by the presented content rather than the method itself.

A second question that was targeted in the present study is whether an illusory slowing down of own bodily movements in VR would affect the speed of the perceived time flow or the expectations regarding the duration of other physical processes (as water pouring out of a bottle). Our results did not reveal a significant difference between the VR conditions including normal versus reduced movement speed, neither in the numerical-unit task nor in the physical-process task. There are many studies showing effects of external *zeitgeber* on timing performance (Landeck et al., 2020; Liu et al., 2021; Schatzschneider et al., 2016). The manipulation of external cues to induce the illusion of a changed speed of time differs in an important way from the manipulation of the speed of own body movements, as it is implemented in the present study. Self-produced bodily movements generate an efference copy which serve as a forward model to predict the sensory consequences of movements (Blakemore et al., 1998; Miall and Wolpert, 1996). This efference copy should enhance the salience of the differences between real and virtual environments: Distorted visual feedback about own body movements in VR are relatively easy to detect on the basis of a comparison with the efference copy, while such an internal standard is not available for external *zeitgeber* stimuli (e.g., an accelerated or decelerated sunrise in Landeck et al., 2020, and Schatzschneider et al. 2016). According to this interpretation, the experimental manipulation in the present study (i.e., a deceleration of the sensory visual effects of own head movements) was easily identified as incorrect information and therefore not taken into account for the time judgments.

Nevertheless, it seems plausible to assume that the timing of bodily movements should have a larger effect on perceived time than the timing of external *zeitgeber*. Many studies highlight the influence of bodily actions and action representations on the sense of time (Gavazzi et al., 2013; Nather et al., 2011, 2015; Nather and Bueno, 2011; Tomassini and Morrone, 2016), and some posit interoceptive states as a basis of time perception (Cohen, 1981; Craig, 2009; Wittmann, 2009; Vohs and Schmeichel, 2003). By using VR, Bansal et al. (2019) manipulated the speed of events in a VR game as a function of moving speed of the participants’ hands, that is, when participants moved their hands faster, they experienced the physical events in VR happening with increasing speed. Adaptation to this situation resulted (relative to pre-adaptation measures) in an underreproduction of time intervals of the range of 3-6 s. This study suggests that a coupling of own body movements with the perceived speed of time in VR can indeed affect the performance in time perception tasks. As Bansal et al. (2019) did not induce a conflict between own body movements and visual feedback regarding these movements, it is also in line with our interpretation of the absence of an effect of movement speed in the present study.

Finally, it is worth noting that the considerations about time perception in VR made here bear an analogy to the domain of space (e.g., Creem-Regehr et al., 2022) and other perceptual qualities like color (e.g., Pardo et al., 2018). There is evidence for a systematic bias in the judgment of spatial distances, which appear shorter when presented in VR as compared to the real world (Creem-Regehr et al., 2022; Kelly, 2022a; Kelly et al., 2022b; Plumert et al., 2005). This phenomenon is often referred to as distance misperception, but the prevailing interpretation is that the presentation in VR (e.g., via head-mounted displays; Kelly, 2022a) causes objects to appear visually closer, rather than presuming a change in the underlying sense of space.

## Conclusion

In the present study we show that VR-induced changes in timing behaviour are restricted to temporal judgments of intervals referring to physical processes, whereas judgments of intervals defined by numerical units are unaltered. This finding confirms an important prerequisite for the use of VR techniques in the study of time perception and strengthens the logical basis for the interpretation of changed timing behaviour induced by the manipulations within VR.

## Data Availability

Raw data and analysis scripts are available on the Open Science Framework (https://osf.io/gmrfq).

## Declarations

### Open Practices Statement

The data and analysis scripts for the experimental study are available at (https://osf.io/gmrfq). The study was not preregistered.
